# The Decalcification of Cervicothoracic Spinal Metastasis of Breast Cancer Due to Discontinuation of Denosumab: A Case Report

**DOI:** 10.7759/cureus.28699

**Published:** 2022-09-02

**Authors:** Masaki Tatsumura, Takeshi Saito, Hiroyuki Ito, Kousei Miura, Masashi Yamazaki

**Affiliations:** 1 Department of Orthopaedic Surgery and Sports Medicine, Tsukuba University Hospital Mito Clinical Education and Training Center / Mito Kyodo General Hospital, Mito, JPN; 2 Department of Breast Surgery, Tsukuba University Hospital Mito Clinical Education and Training Center / Mito Kyodo General Hospital, Mito, JPN; 3 Department of Dental Surgery, Tsukuba University Hospital Mito Clinical Education and Training Center / Mito Kyodo General Hospital, Mito, JPN; 4 Department of Orthopaedic Surgery, Faculty of Medicine, University of Tsukuba, Tsukuba, JPN

**Keywords:** spinal instrumentation, quadriplegia, bone modified agent:, medication-related osteonecrosis of the jaw, breast cancer, adjuvant therapy, recalcification, decalcification, discontinuation of denosumab, metastatic spinal tumor

## Abstract

Breast cancers frequently metastasize to bone. Several guidelines recommend denosumab to control metastasis. In the current case, denosumab allowed the calcification of cervicothoracic spinal metastases following bone decalcification by breast cancer. Six years after administration, denosumab was discontinued and the metastatic lesions became decalcified, but recalcification occurred after re-administration of denosumab. There were no reports of serious decalcification after discontinuation of denosumab.

The patient was a 71-year-old woman who was unable to walk independently because of a fracture of the seventh cervical vertebra and severe spinal cord compression. After immobilization with a halo vest, posterior fixation was performed.

Examination of the pathology of the breast and cervical spine revealed ductal carcinoma of the breast. After docetaxel for four months, tegafur-gimeracil-oteracil potassium (TS-1) was administered and monthly denosumab was initiated. CT showed postoperative recalcification of the cervicothoracic spine, and MRI revealed spinal cord decompression.

The first occurrence of medication-related osteonecrosis of the jaw (MRONJ) occurred five years after cervicothoracic spinal surgery and the second occurrence of MRONJ occurred after six years. Denosumab was discontinued and TS-1 was resumed four months after discontinuation.

Fourteen months after discontinuation of denosumab, the patient felt muscle weakness in the right upper extremity and numbness in both hands. CT showed cervicothoracic spine decalcification and MRI showed spinal cord compression. As there were no signs of recurrence in the primary lesion around the left breast, TS-1 was continued and denosumab was resumed. Three months after the re-administration of denosumab, CT showed recalcification and recovery of upper extremity muscle strength, and MRI revealed improvement in spinal cord compression.

## Introduction

It is widely known that bone-modifying agents (BMAs), such as denosumab and zoledronate, suppress skeletal-related events in metastatic bone tumors [1,2]. Denosumab is highly effective in reducing bone pain, hypercalcemia, and pathological fractures, and is frequently used for metastatic bone tumors. Metastases of solid tumors are known to recur often. Therefore, denosumab discontinuation following complete or good partial response is not recommended currently [2]. On the other hand, drug-induced side effects may require discontinuation of denosumab. Many reports since 2003 show that BMAs can cause medication-related osteonecrosis of the jaw (MRONJ) [3]. Temporary discontinuation is necessary due to oral surgical treatment of MRONJ. Denosumab causes calcification of spinal metastases following decalcification from breast cancer. After discontinuation of denosumab for the treatment of MRONJ, the metastases decalcified but recalcification occurred after re-administration of denosumab.

## Case presentation

A 71-year-old woman with no significant medical background presented with neck pain and progressive upper extremity numbness and weakness. When she was admitted to our institute three months after the onset of her symptoms, she was unable to walk independently. On physical examination, her muscle power assessment (right/left) showed 5/5 strength in all muscle groups tested, except for 4/4 dorsal interossei muscles, 4/2 iliopsoas muscles, 4/3 quadriceps muscles, 5/2 tibialis anterior muscles, 5/2 extensor hallucis longus muscles, and 4/4 extensor digitorum muscles. Sensation was decreased on the anterior surface of the thighs and right forearm. Deep tendon reflexes of the biceps, triceps, brachioradialis, and patellae were normal bilaterally, and she denied bowel or bladder dysfunction. Her neurological status was C2 on the modified Frankel classification [4] and her preoperative Japanese Orthopaedic Association (JOA) score was 6.5/17.

**Figure 1:**
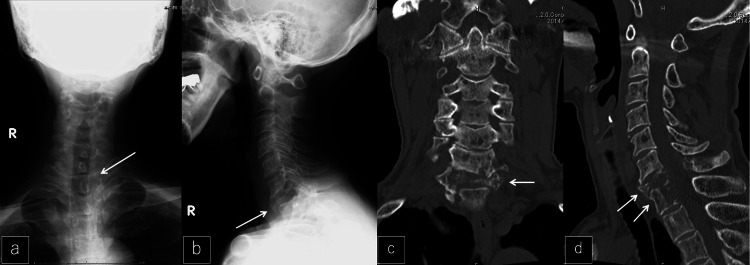
X-ray and CT imaging at the first visit a, b: Preoperative anteroposterior and lateral X-ray. The C7 vertebra was collapsed (white arrows). c, d: Coronal and sagittal CT. Decalcified bone in the posterior wall of C7 and T1 vertebrae (white arrows).

 Plain X-rays and computed tomography (CT) revealed a fracture of the seventh cervical vertebra (Figures 1a-1d).

**Figure 2:**
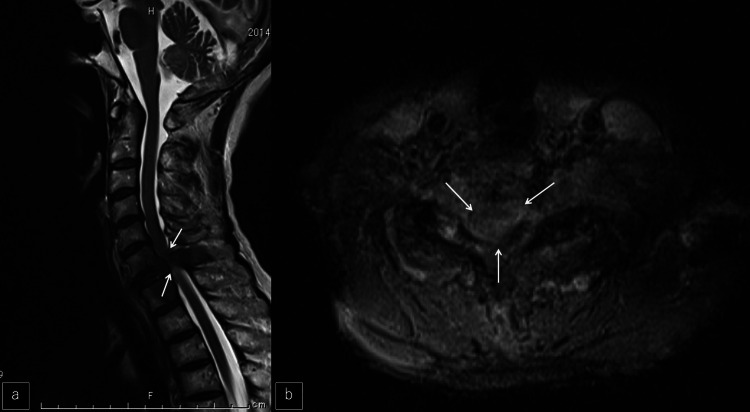
MR imaging at the first visit a, b: Sagittal and axial MRI. A severely compressed spinal cord was shown at the C7 vertebra (white arrows).

Magnetic resonance imaging (MRI) revealed serious spinal cord compression due to metastasis from the sixth cervical vertebra to the first thoracic vertebra (Figures 2a-2b). 

**Figure 3:**
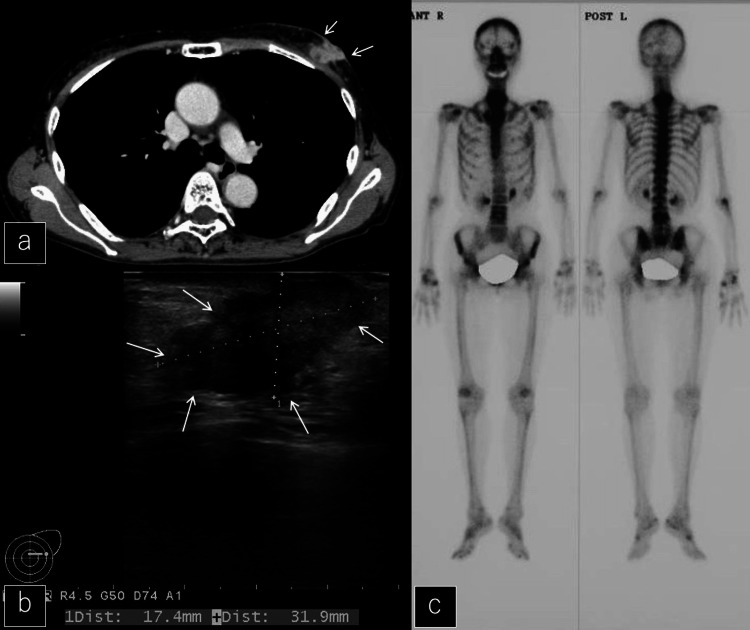
Chest CT, breast ultrasound, and bone scintigram after the first visit a: Chest CT showed an abnormal shadow around a left breast mass (white arrows). b: Ultrasound showed a left mammary tumor (white arrows). c: Bone scintigrams (99mTc) showed multiple bone metastases.

A chest CT showed an abnormal shadow around a left breast mass (Figure 3a) and an ultrasound showed a left mammary tumor (Figure 3b). Bone scintigrams (99mTc) showed multiple bone metastases, including hyperaccumulation in the cervicothoracic spine (Figure 3c).

**Figure 4:**
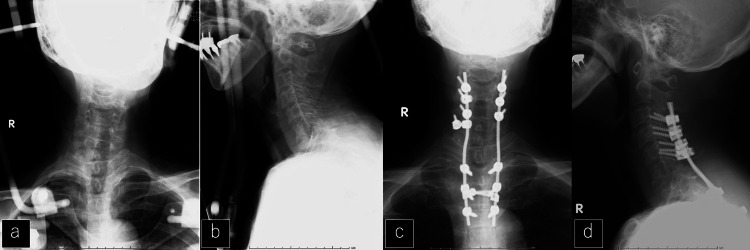
X-ray after halo vest immobilization and posterior fixation a, b: X-ray after temporal halo vest immobilization. c, d: X-ray after posterior fixation.

Her neurological status worsened rapidly and a halo vest was used for immobilization beginning on her initial visiting day to prevent the progression of neurological deficits (Figures 4a-4b). After several days, a fine-needle aspiration biopsy was performed and posterior instrumented fixation without decompression was completed from the third cervical vertebra to the fourth thoracic vertebra, accompanied by an open biopsy (Figures 4c-4d).

**Figure 5:**
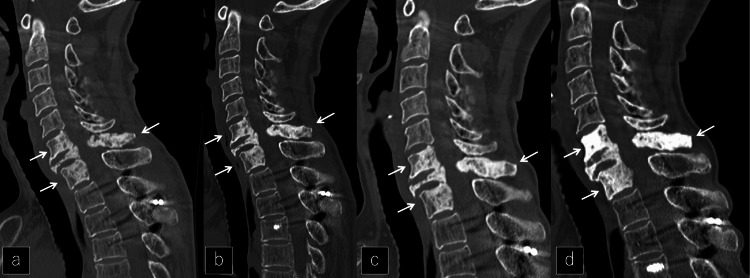
Changing image over time on CT for five years a, b, c, d: Sagittal CT at 3 months, 1 year, 2 years, and 5 years. Sclerotic change progressed in the affected vertebrae (white arrows).

**Figure 6:**
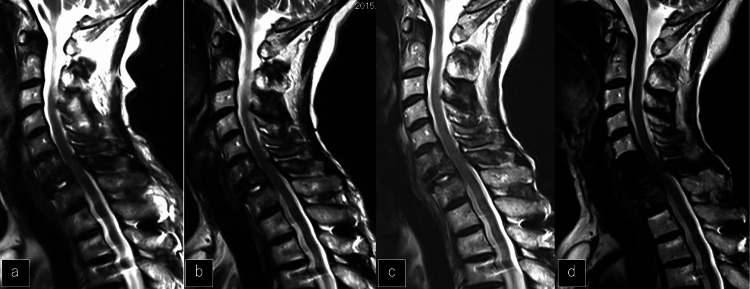
Changing image over time on MRI for five years a, b, c, d: Sagittal MRI at 3 months, 1 year, 2 years, and 5 years. Spinal cord decompression was maintained for 5 years.

The pathology results of the breast needle biopsy and cervical spine indicated invasive ductal carcinoma of the breast. We diagnosed it as a ductal carcinoma of the breast (T4cN1M1 (oss) StageⅣ). The immunohistochemical diagnosis was the triple-negative type, indicating that human epidermal growth factor receptor-2 (Her2) and estrogen and progesterone receptors also were negative. The patient was judged to be a candidate for chemotherapy because of triple-negative disease. Chemotherapy with docetaxel (DTX) was started and resulted in a marked reduction of the left breast tumor. Therefore, six courses of 70 mg/m2 of DTX over 18 weeks were completed. After DTX, 100 mg/day of tegafur-gimeracil-oteracil potassium (TS-1) was administered from four months to five years postoperatively. Adjuvant subcutaneous denosumab, 60 mg monthly, was started. CT showed recalcification of the vertebrae of C6-T1 at three months (Figure 5a), one year (Figure 5b), two years (Figure 5c), and five years (Figure 5d) postoperatively; MRI revealed the release of spinal cord compression by the tumor (Figure 6a) and kept the release of spinal cord compression (Figures 6b-6d).

The neurological improvement to a modified Frankel grade D3 was obtained two weeks after cervicothoracic surgery and the postoperative JOA score of cervical myelopathy score improved to 15.5/17.

As no organs other than bone, and the disease, were controlled by TS-1, a left mastectomy and axillary lymph node dissection were performed to control the primary lesion three years after cervicothoracic spine surgery.

**Figure 7:**
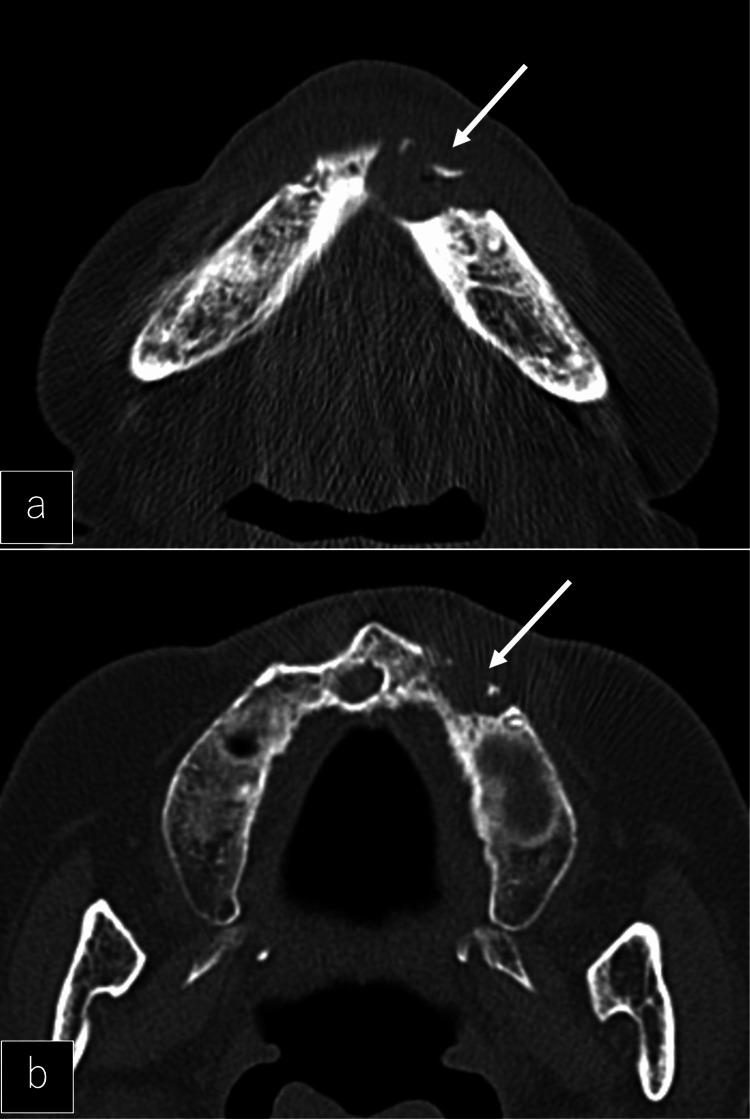
Necrosis of the jaw on CT a: Axial CT showed a bone translucency spot on the left mandible (white arrow). b: Axial CT showed a bone translucency spot on the left maxilla (white arrow).

The first sign of MRONJ occurred in the left mandible five years after cervicothoracic spine surgery. Denosumab was stopped three months before the oral surgery and TS-1 was stopped one month before. MRONJ was cured with conservative therapy after the first oral surgery. Denosumab and TS-1 were resumed one month after the oral surgery. MRONJ of the left mandible recurred six years after cervicothoracic spine surgery(Figure 7a) but also included MRONJ of the left maxilla (Figure 7b).

**Figure 8:**
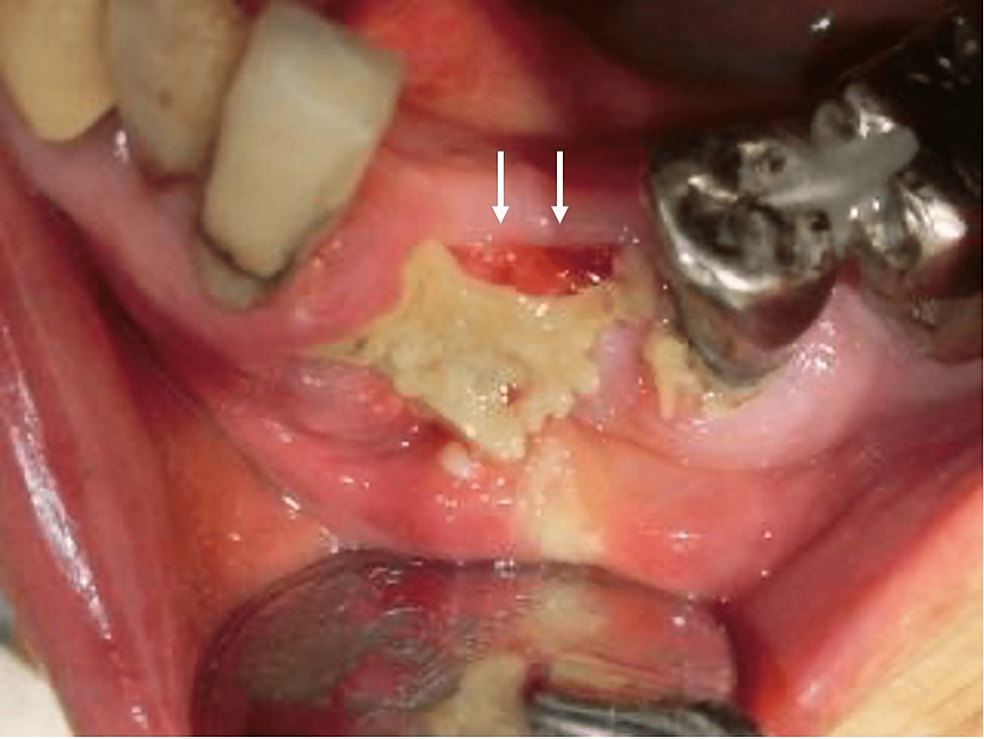
Photograph of gingiva on necrosis of the jaw Photograph of the superficial layer of the left mandibular necrotic area (white arrows).

The lesion was more serious than the previous necrosis (Figure 8), and surgical therapy was chosen.

**Figure 9:**
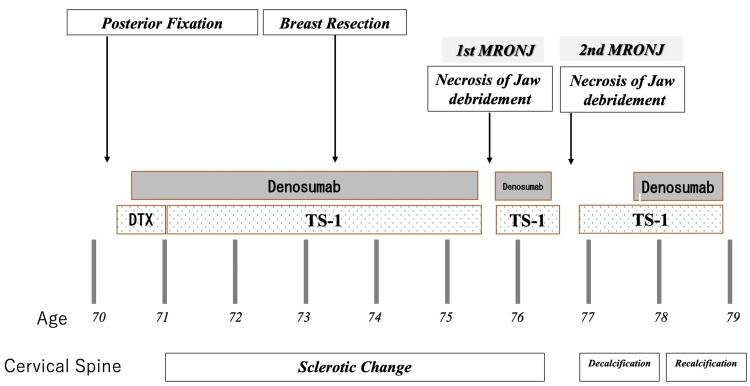
Medication history Decalcification and recalcification were linked with the discontinuation and resumption of denosumab. (DTX; docetaxel, TS-1; tegafur-gimeracil-oteracil potassium)

Denosumab was stopped for three months before the second oral surgery. TS-1 was stopped one month before surgery and restarted five months after discontinuation. Conservative dental treatment was continued after surgery, and discontinuation of denosumab was continued (Figure 9).

**Figure 10:**
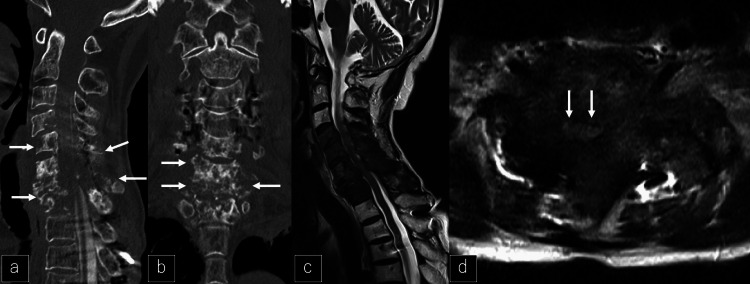
CT and MRI after discontinuation of denosumab a, b: Sagittal and coronal CT one year after discontinuation of denosumab showing bone decalcification from C4-T1 (white arrows). c, d: Sagittal and axial MRI one year after discontinuation of denosumab showing spinal cord compression (white arrows).

Fourteen months after the last discontinuation of denosumab, the patient could not use chopsticks. Muscle weakness in the right upper extremity and numbness in both hands also appeared. A myelogram CT showed bone decalcification from C4-T1 (Figures 10a-10b) and MRI showed spinal cord compression (Figures 10c-10d).

**Figure 11:**
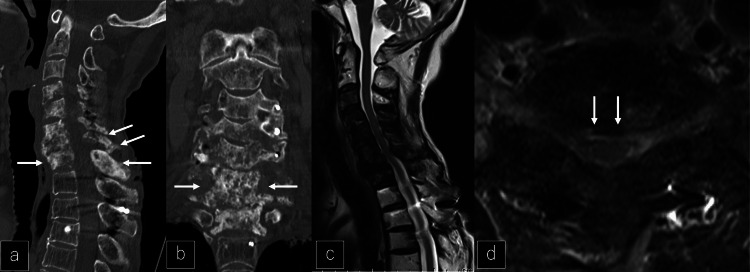
CT and MRI after the resumption of denosumab a, b: Sagittal and coronal CT after the resumption of denosumab showing bone recalcification from C4-T1 (white arrows). c, d: Sagittal and axial MRI after the resumption of denosumab showing the release of spinal cord compression (white arrows).

As there was no sign of recurrence in the primary lesion around the right breast, TS-1 was continued without any change. Treatment of MRONJ continued but denosumab administration was resumed. Three months after resuming denosumab, the CT showed recalcification (Figures 11e-11f) and recovery of upper extremity muscle strength. MRI also revealed reduced compression around the spinal cord (Figures 11g-11h).

## Discussion

Prior to osteonecrosis of the jaw, the patient had recovered from paralysis after cervicothoracic spine surgery, bone calcification was resumed with denosumab, and both the primary and metastatic lesions appeared to have resolved. Bone decalcification did not occur during the first episode of MRONJ following denosumab discontinuation, perhaps because of the short duration of discontinuation. The effects of denosumab on bone are mostly diminished within six months of treatment discontinuation [5]. We do not believe that withdrawal of the drug within six months will affect calcium reabsorption.

The second episode of MRONJ was severe enough to require surgery and prolonged denosumab discontinuation. Despite continued TS-1, the decalcification of the vertebrae occurred and the metastatic tumor recurred. The only drug involved in the recalcification of bone, in this case, was the resumption of denosumab. Despite the fact that no other conditions were altered, discontinuation of denosumab caused marked decalcification, and resumption of denosumab caused rapid recalcification.

It is well known that breast cancers frequently metastasize to bone, including vertebrae. Generally, spinal metastasis accompanied by vertebral pathological fractures resulting in neurologic deficits requires surgical decompression and posterior fixation [6]. In patients with tumor-related spinal instability, posterior stabilization is controlled by installing instrumentation. In this present case, we perform only posterior fixation without decompression because the neurologic symptoms were recovered by temporary halo immobilization. In addition, the promotion of fracture healing and bone hardening reduces the risk of implant failure around the fixed lesion. BMA administration is expected to improve postoperative outcomes due to increased bone strength that promotes bone recalcification. On the other hand, osteonecrosis of the jaw, which is a major complication, is known to increase in frequency with prolonged denosumab administration, with a frequency of up to 18% in patients with carcinoma.

There have been other reports of problems with discontinuation of denosumab, including hypercalcemia [7], rebound osteoporosis [8], and fractures [2], especially vertebral [9] and femoral neck fractures [10].

Unfortunately, this patient had to discontinue denosumab due to the treatment of MRONJ. We proposed that the prolonged discontinuation caused decalcification, spinal instability, and recurrence of neurological symptoms. After the resumption of denosumab administration, the patient had quick recalcification of the spine and recovered from her neurological symptoms. In this case, the implant became an MRI artifact and spinal cord evaluation was difficult, so implant removal sometimes is advised when cervical lesions are stabilized due to osteosclerosis of the vertebrae. Considering that marked bone resorption can occur as in the present case, we suggest that implant removal not be performed even in apparently improved cases.

If the malignancy is not well controlled, spinal metastases can worsen and cause paralysis even after surgery. Systemic treatment is necessary to control the disease so that paralysis does not occur postoperatively. Similarly, systemic administration of denosumab to control calcification should be done with caution and with consideration for side effects.

## Conclusions

A metastatic cervicothoracic lesion from breast cancer, which appeared to be cured, was decalcified with neurological symptoms one year after denosumab discontinuation. After the resumption of denosumab, the lesion quickly recalcified and neurological symptoms resolved. Even long after treatment is initiated, withdrawal of denosumab results in marked destruction of bone from metastasis, and resumption of denosumab allows the bone to regenerate.
